# Refining the diagnosis of acute heart failure in the elderly

**DOI:** 10.1002/ehf2.15017

**Published:** 2024-08-12

**Authors:** Mauro Riccardi, Matteo Pagnesi

**Affiliations:** ^1^ Institute of Cardiology, ASST Spedali Civili, Department of Medical and Surgical Specialties, Radiological Sciences and Public Health University of Brescia Brescia Italy

Natriuretic peptides (NPs) have a central role in the diagnosis of acute heart failure (AHF) as stated by the latest guidelines and recent clinical consensus statements.^1^, ^2^, ^3^ NPs should be measured in all patients presenting with symptoms suggestive of new‐onset or worsening HF, particularly dyspnoea, since their use facilitates both early diagnosis and rule‐out of AHF, and serial NP measurement may also be useful after discharge in patients with AHF.^1^, ^3^, ^4^ N‐terminal pro‐B‐type natriuretic peptide (NT‐proBNP) is the most widely used NP in HF since it has a longer half‐life than brain natriuretic peptide (BNP), resulting in higher concentrations over time, is stable at room temperature facilitating measurement in clinical laboratories and is not affected by treatments that alter BNP degradation (e.g., sacubitril/valsartan).^3^ The use of NPs in the diagnosis of AHF has changed over time: while the first HF guidelines focused only on identifying a cut‐point to rule‐out AHF,^5^ the latest guidelines discuss in parallel both rule‐out and rule‐in cut‐points and also suggest post‐discharge intensive care based on serial NPs measurement.^1^, ^6^ Actually, ideal rule‐in strategies are based on the use of NT‐proBNP age‐adjusted cut‐points, as shown by previous studies: ≥450 pg/mL for patients under 50 years, ≥900 pg/mL for patients aged 50–74 years and ≥1800 pg/mL for patients ≥75 years.^7^ However, as life expectancy increases, gaps remain in the optimal NPs thresholds for HF diagnosis among elderly patients, such as those over 75 years of age or, particularly, those over 85 years of age. These older patients typically have multiple comorbidities that can also alter NP values or modify the interpretation of NP thresholds, such as atrial fibrillation (AF), obesity and renal failure.^8^, ^9^, ^10^, ^11^

In this issue of the ESC Heart Failure, Berthelot et al. report the results of a retrospective, multicentre, real‐world cohort study testing the optimal NPs diagnostic thresholds for the diagnosis of AHF in patients aged over 75 years.^12^ In this contemporary cohort (study period 2011–2022), 12 071 hospitalized patients with an initial emergency department (ED) admission for acute dyspnoea and with available NT‐proBNP or BNP measurement within 48 h since admission were included. According to International Classification of Disease (ICD)‐10 codes, a diagnosis of AHF was observed in 66% (*n* = 7946) of included patients, and respiratory dyspnoea was the diagnosis in the remaining 35% (*n* = 4125). Compared with patients with other causes of dyspnoea, AHF patients had higher NT‐proBNP levels (5021 vs. 832 pg/mL, *P* < 0.01). The identified optimal overall NT‐proBNP threshold for diagnosing AHF was 1748 pg/mL, with a positive predictive value (PPV) of 84%. Stratifying the cut‐points by age, among patients aged 75–85 years a threshold of 1680 pg/mL was associated with a PPV of 86%, while in patients over 85 years a cut‐point of 2235 pg/mL had a PPV of 84%. Moreover, analysing the various subgroups based on the presence of comorbidities, in elderly patients with AF the most appropriate NT‐proBNP threshold was 2332 pg/mL, while in patients with an estimated glomerular filtration rate <30 mL/min, the optimal NT‐proBNP threshold was 3474 pg/mL, both yielding a 90% PPV for AHF diagnosis. Lastly, in patients with obesity, a threshold of 1375 pg/mL had an 85% PPV for AHF diagnosis.^12^

The main limitation reported by the author is that the diagnosis of AHF was made by physicians through ICD‐10 also based on NPs levels at admission, without independent adjudication at discharge. This bias might have influenced the assessment of the diagnostic accuracy of NPs in this study. Furthermore, the retrospective design with other potential biases, the lack of echocardiographic data and the potential overestimation of AHF prevalence in this cohort (due to exclusion of patients without available NP measurement) might also have influenced the study findings. For example, further assessment of NP cut‐offs in older patients with HF with reduced vs. preserved ejection fraction or older patients with concomitant valvular heart diseases would be of great interest.

Nevertheless, the authors should be congratulated for this large study exploring the role of NPs in this complex subset of patients (older and with comorbidities), where timely AHF diagnosis is challenging and of utmost importance. As previously demonstrated,^7^ age‐stratified cut‐points for NPs increase with age. In this study, the 1800 pg/mL NT‐proBNP cut‐off for patients over 75 years was essentially confirmed (optimal threshold of 1748 pg/mL), and the authors also identified a 2235 pg/mL threshold with good PPV in patients over 85 years, thus providing data on NP diagnostic usefulness in the very elderly. Importantly, the PPV in patients ≥75 years outperformed the 62% value reported in the ICON‐RELOADED study.^13^ Practical and validated rule‐in NP cut‐points are crucial for timely and accurate diagnosis of AHF already in the ED, thus allowing to immediately start treatment. Similarly, rule‐out of AHF may be particularly challenging in older patients, as demonstrated by the recently developed CoDE‐HF (Collaboration for the Diagnosis and Evaluation of Heart Failure) decision support tool that had a negative predictive value of 94.6% using the rule‐out NT‐proBNP cut‐point ≤300 pg/mL in the overall cohort, but with a reduction to 88.2% in patients over 75 years.^14^

Although a recent clinical consensus statement suggested that the age‐adjusted rule‐in NT‐proBNP values for early HF diagnosis in the ED do not necessitate additional adjustments for factors that can influence NP levels,^3^ this study found significantly higher optimal NP cut‐points in older patients with AF and especially renal failure.^12^ This finding underlines the importance of assessing and considering comorbidities in older patients with suspected AHF, also to refine the interpretation of NP values. The issue of identifying increasingly precise, age‐adapted NT‐proBNP thresholds, while maintaining a rule‐out cut‐point <300 pg/mL in all patients, is to expand the grey area where NP measurement alone cannot be sufficient to achieve a reliable rule‐in or rule‐out of AHF in older patients (>75 years). In this area, about 50% of patients will have a diagnosis of AHF with intermediate mortality compared to patients with NT‐proBNP values above the rule‐in or below the rule‐out thresholds.^15^ Additional tests, such as chest X‐ray plus echocardiography, along with clinical judgement become crucial for a proper AHF diagnosis in these grey zones.

The assessment of NPs remains critical in the process of diagnosing AHF, with the aim of obtaining an early rule‐out or rule‐in. However, NPs remain non‐specific for HF diagnosis in several scenarios, such as comorbidities that may result in increased NP levels or other conditions associated with lower‐than‐expected NP levels. These challenging scenarios are more common in patients over 75 years, particularly in the very elderly. Along with individualized interpretation of biomarker levels, judicious clinical assessment and additional diagnostic tests are very useful in this setting to confirm or exclude AHF. In this context, the study by Berthelot et al. helps in refining the interpretation of NP values in older patients, also providing insights in the very elderly or in patients with relevant comorbidities. However, future research is needed to further refine the use of NPs and, eventually, the art of diagnosing HF in older and comorbid patients.

## Conflict of interest

Dr. Pagnesi has received personal fees from Abbott Vascular, AstraZeneca, Boehringer Ingelheim, Novartis, Roche Diagnostics and Vifor Pharma. Dr. Riccardi has no conflicts of interest to disclose.
